# PubAnnotation-query: a search tool for corpora with multi-layers of annotation

**DOI:** 10.1186/1753-6561-9-S5-A3

**Published:** 2015-08-06

**Authors:** Jin-Dong Kim, Kevin Bretonnel Cohen, Jung-jae Kim

**Affiliations:** 1 Database Center for Life Science, Research Organization of Information and Systems, Kashiwa, Japan; 2 School of Medicine, University of Colorado, Denver, Colorado, US; 3 School of Computer Engineering, Nanyang Technological University, Singapore

## Summary

PubAnnotation provides a convenient platform to collect and align corpora with various annotations. However, corpora must be searchable to be useful, but there has been no standard way to search corpora, particularly when multiple layers of annotations are present. *PubAnnotation-query *is designed to provide an interface for searching corpora annotated with multiple layers. This paper describes the tool, with some example use cases. Its use is illustrated with two separate corpora.

## Introduction

*PubAnnotation *[1] provides a convenient platform to collect and align corpora with various annotations. However, corpora must be searchable to be useful, but there has been no standard way to search corpora, particularly when multiple layers of annotations are present. *PubAnnotation-query *is designed to provide an interface for searching corpora annotated with multiple layers. It is based on *RDF *and *SPARQL*, which is an emerging standard of data representation and search framework, particularly for the Web environment.

Representing linguistic data in RDF is a growing area of linguistic research [2,3]. Verspoor and Livingston point out that a number of advantages that accrue from representing the annotations in corpora as RDF, including interoperability, information sharing and reuse, Web-scale collaboration and analysis, and availability of tools [3]. Verspoor and Livingston review the DOMEO and Utopia Document [4] tools. These tools have in common the goal of allowing semantic representation and visualization of linguistic (and other) annotations. What has been missing from the tools landscape is a tool that would allow searching of corpora with annotations. If a corpus is to be useful for linguistic research, it must be searchable. The work described here led to the development of *PubAnnotation-query*, a tool for searching such corpora. It allows for searching multiple layers of annotation, using SPARQL, a standard search language of semantic web.

## Context and related works

Corpora and corpus search tools can be thought of as having been developed in an environment of co-evolution. Early corpora, often with only part-of-speech annotation, led to the development of *Keyword In Context (KWIC) *tools, or concordancers [5,6]. *Penn Treebank *[7] became useful for linguistic research with the development of *tgrep. PropBank *[8] is accessible through the Unified Verb Index [9]. The *Sketch Engine *[6] holds the promise of revolutionizing corpus linguistics by the fact that it makes unprecedented numbers of corpora searchable through a single interface. To date, there has been no search interface available for multi- layered annotation in RDF (or, to our knowledge, any demonstration that it is even feasible). The work reported here aims to remedy that situation.

## Materials and methods

### Materials

To develop and validate *PubAnnotation-query*, two corpora were converted to RDF. The *CRAFT *corpus consists of 560,000 words of manually annotated text, containing annotations of document structure, *Penn*-style tree banking, and seven classes of named entities [10,11]. The *GRO *corpus consists of 200 *PubMed *abstracts of manually annotated text, containing annotations for 10,395 named entities and events [12].

### Methods

The structural, syntactic, and named entity annotations of CRAFT and the event annotations of GRO were converted to RDF. For the RDF representation, *Text Annotation Ontology (TAO)*, an original vocabulary for text annotation, was designed with a particular focus on enabling search. Consequently, the searching mechanism implemented in *PubAnnotation-query *makes use of SPARQL queries. Development of the provided functionality was informed by the following use cases:

• In order to create a lexical resource, discover selectional restrictions on arguments of a predicate.

• In order to write a grammar, find examples of subcategorization frames.

• In order to write event extraction patterns, find example events of given types and trigger words.

These use cases require searching across multiple layers of annotation, in particular, syntax, terminal strings, and named entities. To direct the development task, specific sets of searches were developed. These were divided into single-layer and multi-layer searches. As conceived of in this project, single-level searches target a word (*find all sentences containing the word 'bind'*), a lemma (*find all sentences containing any form of 'bind'*), a syntactic construction (*find all sentences or phrases containing a verb phrase that dominates two noun phrases*), or a named entity (*find all sentences containing a Sequence Ontology annotation*). Multi-level searches require searching for some combination of these, such as a word and a named entity (*find all sentences containing 'bind' followed by a Sequence Ontology annotation*), lemma plus term (*find all sentences containing any form of bind followed by a Sequence Ontology annotation*), syntax plus named entity (*find all verb phrases dominating any named entity, find all verb phrases in which an argument of the verb is a named entity*).

## Results

**Figure 1:**
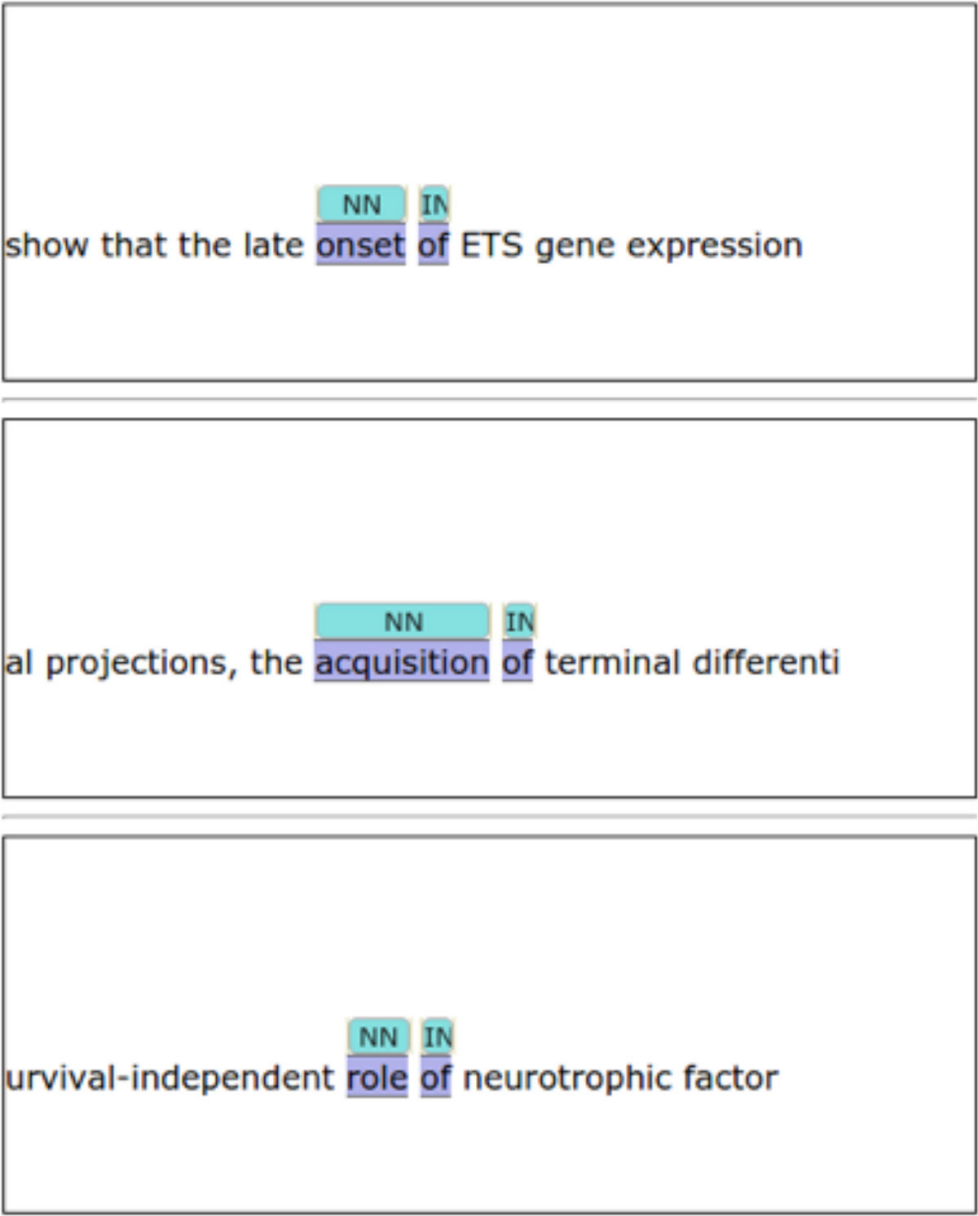
**Example of linguistic pattern search**.

A preliminary version of *PubAnnotation-query *is implemented and made publicly available at http://query.pubannotation.org/ for a proof-of-concept. As an example, the following SPARQL query tells it to find two consecutive spans of *NN *and *IN *where the lexical value of the *IN *is *of *. Figure 1 shows a fraction of the results.

PREFIX penn:<http://example.org/penntag.owl#>

PREFIX tao:<http://pubannotation.org/ontology/tao.owl#>

       select ?s1 ?s2 where {

       ?o1 a penn:NN; tao:denoted_by ?s1.

       ?o2 a penn:IN; tao:denoted_by ?s2.

       ?s1 tao:part_of ?t1;

       tao:ends_at ?p1.

       ?s2 tao:part_of ?t2; tao:begins_at ?p2; tao:has_value "of".

       FILTER (?t1 = ?t2) FILTER (?p1 + 1 = ?p2)

} limit 100

## Limitations and future directions

Although TAO is designed with a focus to enable search over multi-layers of annotation, composing search queries for *PubAnnotation-query *may be still difficult to non-experts, and follow-up efforts for easing the query composition is necessary. To benefit from the interoperability of semantic web, compatibility with other existing corpus annotation frameworks also need to be explored.
